# Synthesis, Characterization and Biocidal Activities of Some Schiff Base Metal Complexes

**DOI:** 10.4103/0250-474X.65015

**Published:** 2010

**Authors:** M. A. Neelakantan, M. Esakkiammal, S. S. Mariappan, J. Dharmaraja, T. Jeyakumar

**Affiliations:** Chemistry Research Centre, National Engineering College, K. R. Nagar, Kovilpatti-628 503, India; 1 Chemistry Section, Faculty of Engineering and Technology, Annamalai University, Annamalai Nagar-608 002, India

**Keywords:** Antibacterial activities, antifungal activities, mixed ligand complexes, *o*-vanillin, schiff base, spectral studies

## Abstract

Some new mixed ligand complexes (1-5) of type ML'B (M(II)=Mn(II), Co(II), Ni(II), Cu(II) and Zn(II); HL'= o-vanillidene-2-aminobenzothiazole; B= 1,10-phenanthroline) and Schiff base metal complexes of types (ML_2_") (6-10) and (M_2_L") (11-15) (HL"= *o*-vanillidene-2-amino-N-(2-pyridyl)-benzene sulfonamide) were synthesized and characterized by elemental analysis and spectral (IR, ^1^H NMR and ^13^C NMR) studies. The free ligands and their metal complexes have been screened for their *in vitro* biological activities against bacteria, fungi and yeast. The metal complexes show more potent activities compared with Schiff base ligands.

Metal complexes of N and S chelating ligands have attracted considerable attention because of their interesting physicochemical properties and pronounced biological activities. The N and S atoms play a key role in the coordination of metals at the active sites of numerous metallobimolecules. *o*-vanillin is a natural aldehyde found in *Andropogen nardus*. It is used to treat bellyaches and also used in spicery[1]. Schiff bases containing *o*-vanillin possesses antifungal, antibacterial properties[2] and it acts as a weak inhibitor of tyrosinase, display both antimutagenic and comutagenic properties in *Escherichia coli*[3]. Heterocycles containing thiazole ring is present in a number of pharmacologically and biologically active compounds. Compounds containing benzothiazole and sulphonamide derivatives were used as antifungal[4,5], antiinflammatory[6], antiHIV[7], anticancer[8], anticarbonic anhydrase[9], diuretic, hypoglycaemic[10], antithyroid[11], antimalarial and in therapeutic fields. In view of the pronounced biological activities of these compounds, we report herein the synthesis and characterization of (i) mixed ligand complexes of Mn(II), Co(II), Ni(II), Cu(II) and Zn(II) derived from 1,10–phenanthroline and *o*-vanillidene-2-aminobenzothiazole (HL') and (ii) Schiff base metal complexes of *o*-vanillidene-2-amino-N-(2-pyridyl)-benzene sulfonamide (HL"). The biological screening of free ligands and their complexes against different bacteria, fungi and yeast are reported.

## MATERIALS AND METHODS

All the chemicals and solvents used were of AR grade (Merck, Mumbai, India) except *o*-vanillin (Fluka, Switzerland) and 2-amino-N-(2-pyridyl)-benzene sulfonamide (Sigma, USA). Melting points of all the compounds were determined in open glass capillaries and are uncorrected. The purity of the Schiff bases was ascertained by TLC on Silica gel-G plates and spots were visualized by using iodine vapours. The elemental analysis was performed using a Thermo Finnigan Flash EA 1112 CHNS analyzer at Central Electro Chemical Research Institute (CECRI), Karaikudi, India. The conductometric measurements of the complexes were carried out in DMSO solution using Systronics 611 conductivity bridge. Vibrational spectra were recorded using KBr pellets on FT-IR Shimadzu 8400S spectrophotometer, in the region 4000 – 400 cm^-1^ range. The ^1^H NMR and ^13^C NMR of Schiff base ligands and their diamagnetic zinc complexes in DMSO-d_6_ were recorded on Perkin Elmer R-32 spectrometer using Tetramethylsilane as internal standard at IIT, Chennai, India.

## Biological evaluation:

The newly synthesized ligands and their metal complexes were screened *in votro* for their antibacterial activity against bacteria: *Escherichia coli, Pseudomonas aeruginosa, Salmonella Typhi and Vibrio parahaemolyticus* by well diffusion method[12] using agar nutrient. The antifungal activities were tested against fungus: *Aspergillus Niger, Penicillium, Trichoderma virida* and yeast: *Saccharomyces cerevisiae* by well diffusion method using potato dextrose agar as the medium. Ampicillin and nystatin are used as control for bacteria and fungi, respectively. The suspension of each microorganism was added to a sterile agar medium, then poured into sterile Petri plates and left to solidification. The well was dug in the agar media using sterile metallic borer in each plate. The test solution (3×10^-3^ M) was prepared by dissolving the compounds in DMSO and the well was filled with the test solution using micropipette. The plates were incubated for 24 h in the case of bacteria and 72 h for fungi at 35°. The extracts were subjected to further assay with a series on time basis (24, 48 and 72 h). During this period, the test solution was diffused and affected the growth of the inoculated microorganisms. Activity was determined by measuring the diameter of the zone showing complete inhibition (mm). Growth of inhibition was compared with the control. The zone of inhibition is given as the average of three independent determinations.

## Synthesis of mixed ligand complexes (1-5):

**Fig. 1:**
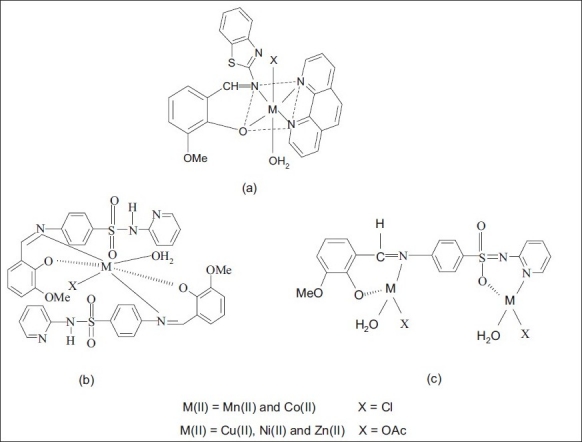
Structure of the complexes. (a) Mixed ligand complexes (1-5), (b) Schiff base complexes (6-10) and (c) Schiff base complexes (11-15).

The Schiff base (HL') was synthesized[13] by the condensation of 20 ml of *o*-vanillin (0.030 g/10 mmol) with 2-amino benzothiazole (0.030 g/10 mmol) in ethanol (1:1 molar ratio). Ten millilitres of Schiff base (0.028 g/10 mmol) and 1,10-phenanthroline (0.020 g/10 mmol) in ethanol was added drop wise to 10 ml of 10 mmol metal salts in hot ethanol (0.020g of MnCl_2_.4H_2_O, 0.024g of CoCl_2_.6H_2_O, 0.025 g of Ni(CH_3_COO)_2_.4H_2_O, 0.020 g of Cu(CH_3_COO)_2_.H_2_O and 0.022 g of Zn(CH_3_COO)_2_. 2H_2_O). The reaction mixture was refluxed for 3 h on a water bath and the volume of the solution was reduced to half of its original volume. The solid compound (fig 1) obtained was filtered off, washed with water, diethyl ether and dried in vacuum over CaCl_2_. The color, elemental analysis data, molar conductivity and melting point of Schiff base (HL') and its mixed ligand complexes (1-5) are given in Table 1.

**TABLE 1:** PHYSIO-CHEMICAL PROPERTIES OF SCHIFF BASE AND METAL COMPLEXES

Compound	Color	Mol. formula^a^	Mol. Wt.	Yield (%)	^m(Ω^-1^ cm^2^ mol^-1^)	Mp (°)
HL'	Reddish yellow	C_15_H_12_N_2_O_2_S	284.00	65	6.78	180
HL"	Orange	C_19_H_17_N_3_O_4_S	383.42	94	4.23	198
1	Pale yellow	MnC_27_H_21_O_3_N_4_SCl	571.74	67	11.38	205
2	Green	CoC_27_H_21_O_3_N_4_SCl	575.73	62	17.39	145
3	Light brown	NiC_29_H_24_O_5_N_4_S	599.04	69	14.22	169
4	Dark brown	CuC_29_H_24_O_5_N_4_S	603.90	67	9.42	202
5	Yellow	ZnC_29_H_24_O_5_N_4_S	605.76	65	6.78	130
6	Brown	MnC_38_H_34_O_9_N_6_S_2_Cl	896.43	54	5.50	210
7	Lignt Brown	CoC_38_H_34_O_9_N_6_S_2_Cl	899.88	64	8.10	>300
8	Lignt Green	NiC_40_H_37_O_11_N_6_S_2_	900.22	59	3.00	285
9	Black	CuC_40_H_37_O_11_N_6_S_2_	904.93	63	5.13	290
10	Dark yellow	ZnC_40_H_37_O_11_N_6_S_2_	906.86	57	3.40	220
11	Light brown	Mn_2_C_19_H_19_O_6_N_3_SCl_2_	718.27	58	9.80	225
12	Brown	Co_2_C_19_H_19_O_6_N_3_SC_l2_	725.17	51	13.90	>300
13	Dark Green	Ni_2_C_23_H_25_O_10_N_3_S	725.85	68	12.90	293
14	Black	Cu_2_C_23_H_25_O_10_N_3_S	663.28	83	10.80	320
15	Light yellow	Zn_2_C_23_H_25_O_10_N_3_S	739.13	56	12.40	254

^a^C, H and N are within the limit of ± 0.3% and S±0.4%; HL'- o-vanillidene-2-aminobenzothiazole; HL"- o-vanillidene-2-amino-N-(2-pyridyl)-benzene sulfonamide

Compound 1, yield: 67%, mp: 205°, IR (KBr) cm^-1^: 1639 (C=N, azomethine), 1557 (C=N, thiazole ring), 1217 (C–O, phenolic), 746 (C–S–C, thiazole ring), 569 (Mn–N), 424 (Mn–O), 3396 and 856 (H_2_ O molecule). Compound 2, yield: 62%, mp: 145°, IR (KBr) cm^-1^: 1618 (C=N, azomethine), 1558 (C=N, thiazole ring), 1215 (C–O, phenolic), 747 (C–S–C, thiazole ring), 572 (Co–N), 430 (Co–O), 3387, 848 (H_2_O molecule). Compound 3, yield: 69%, mp: 169°, IR (KBr) cm^-1^: 1624 (C=N, azomethine), 1558 (C=N, thiazole ring), 1207 (C–O, phenolic), 747 (C–S–C, thiazole ring), 567 (Ni–N), 420 (Ni–O), 3387, 850 (H_2_O molecule). Compound 4, yield: 67%, mp: 199°, IR (KBr) cm^-1^: 1606 (C=N, azomethine), 1560 (C=N, thiazole ring), 1217 (C–O, phenolic), 748 (C–S–C, thiazole ring), 540 (Cu–N), 422 (Cu–O), 3380 and 852 (H_2_O molecule). Compound 5, yield: 65%, mp: 130°, IR (KBr) cm^-1^: 1641 (C=N, azomethine), 1561 (C=N, thiazole ring), 1213 (C–O, phenolic), 748 (C–S–C, thiazole ring), 563 (Zn–N), 437 (Zn–O), 3321, 852 (H_2_ O molecule), ^1^H NMR (TMS, DMSO-d_6_) δ ppm: 7.67 (s, 1H, HC=N, azomethine), 6.80–7.40 (d, 4H, Ar–H), 6.90 (s, 1H, HC=N, thiazole ring), 3.70 (s, 3H, OCH_3_), 1.80 (s, 3H, OOCCH_3_).

## Synthesis of Schiff base:

**Scheme 1:**
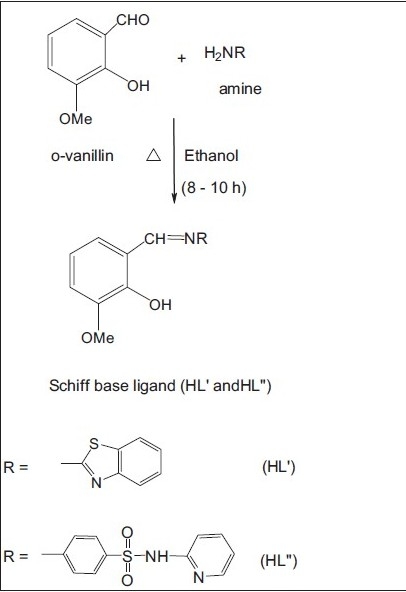
Synthesis of Schiff base (HL' and HL") ligands

The Schiff base (HL") was prepared by refluxing a mixture of equimolar quantities of *o*-vanillin (10 ml, 0.300 g/10 mmol) and 2-amino-N-(2-pyridyl)-benzene sulfonamide (10 ml, 0.490 g/10 mmol,Scheme 1) in hot ethanol. After 8 h of refluxing, the reaction mixture was kept at room temperature overnight and the orange colored product was filtered, washed with distilled water, diethyl ether and recrystallized from the same solvent. Yield: 85%, mp: 198°, IR (KBr) cm^-1^: 1631 (C=N, azomethine), 1269 (C–O, phenolic), 1361, 1138 (SO_2_), 3246 (N–H, sulfonamide group), 1025 (–HC=N, pyridine ring nitrogen), ^1^H NMR (TMS, DMSO-d_6_) δ ppm: 9.92 (s, 1H, HC=N, azomethine), 12.82 (s, –OH), 7.60–8.00 (d, 4H, Ar – H), 8.40–8.60 (s, pyridine –HC=N), 11.04 (s, NH), 3.48 (s, 3H, OCH_3_ ), 2.30 (s, 3H, CH_3_ ), 5.72 (s, 2H, SO_2_ NH group), ^13^C NMR (TMS, DMSO-d_6_) δ ppm: 164.66 (HC=N, azomethine), 155.29 (–C–OH, phenolic), 151.44 (HC=N, pyridine ring), 142.04–140.36 (C=C, pyridine ring), 148.51 (C–O–C), 121.69–117.96 (Ar, C=C), 56.24 (OCH_3_ ).

## Synthesis of metal complexes (6-15):

Ten millilitres of metal salts (10 mmol, 0.02 g of MnCl_2_.4H_2_O, 0.024 g of CoCl_2_.6H_2_O, 0.025 g of Ni(CH_3_COO)_2_.4H_2_O, 0.02 g of Cu(CH_3_COO)_2_. H_2_O and 0.022 g of Zn(CH_3_COO)_2_. 2H_2_O) in hot ethanol was added to 10 ml of the Schiff base ligand (0.038 g/10 mmol) in ethanol (1:1). 10 ml of metal salts (20 mmol, 0.04 g of MnCl_2_ 4H_2_O, 0.048 g of CoCl_2_.6H_2_O, 0.05 g of Ni(CH_3_COO)_2_.4H_2_O, 0.04 g of Cu(CH_3_COO)_2_. H_2_O and 0.044 g of Zn(CH_3_COO)_2_. 2H_2_ O) in hot ethanol was added to 10 ml of the Schiff base ligand (0.038 g/10 mmol) in ethanol (2:1). The mixture was refluxed for 8 h on water bath. The volume of the solution was reduced to one-third of its original volume and left overnight. The precipitate was filtered off, washed with 50% (v/v) ethanol-water mixture, diethyl ether and dried over fused CaCl_2_ in a vacuum desiccator. The elemental analysis, molar conductivity value, color and melting point of Schiff base (HL") and its metal(II) complexes (6-15) are given in Table 1.

Compound 6, yield: 54%, mp: 210°, IR (KBr) cm^-1^: 1599 (C=N, azomethine), 1293 (C–O, phenolic), 1362, 1130 (SO_2_), 3248 (N–H, sulfonamide group), 1026 (–HC=N, pyridine ring nitrogen), 594 (Mn–N), 521 (Mn–O), 3398, 976, 774 (–OH, H_2_O molecule). Compound 7, yield: 64%, mp: >300°, IR (KBr) cm^-1^: 1602 (C=N, azomethine), 1272 (C–, phenolic), 1364, 1132 (SO_2_ ), 3244 (N–H, sulfonamide group), 1030 (C=N, pyridine ring nitrogen), 604 (Co–N), 520 (Co–O), 3405, 976, 759 (–OH, H_2_O molecule). Compound 8, yield: 59%, mp: 285°, IR (KBr) cm^-1^: 1610 (C=N, azomethine), 1286 (C–O, phenolic), 1364, 1135 (SO_2_), 3246 (N–H, sulfonamide group), 1029 (C=N, pyridine ring nitrogen), 588 (Ni–N), 503 (Ni–O), 3398, 968, 767 (–OH, H_2_O molecule), 1616,1402 (–COO, acetate group). Compound 9, yield: 63%, mp: 290°, IR (KBr) cm^-1^: 1616 (C=N, azomethine), 1291 (C–O, phenolic), 1362, 1138 (SO_2_), 3248 (N–H, sulfonamide group), 1027 (C=N, pyridine ring nitrogen), 569 (Cu–N), 513 (Cu–O), 3415, 980, 775 (-OH, H_2_O molecule), 1626, 1412 (–COO, acetate group). Compound 10, yield: 57%, mp: 220°, IR (KBr) cm^-1^: 1598 (C=N, azomethine), 1278 (C–O, phenolic), 1363, 1136 (SO_2_), 3247 (N–H, sulfonamide group), 1026 (C=N, pyridine ring nitrogen), 596 (Zn–N), 507 (Zn–O), 3382, 962, 769 (–OH, H_2_O molecule), 1631,1408 (–COO, acetate group). ^1^H NMR (TMS, DMSO-d_6_) δ ppm: 10.12 (s, 1H, HC=N, azomethine), 7.65–7.97 (d, 4H, Ar–H), 8.38–8.61 (s, pyridine, –HC=N), 11.00 (s, NH), 3.46 (s, 3H, OCH_3_), 2.31 (s, 3H, CH_3_), 5.70 (s, 2H, SO_2_NH group), 4.73 (s, 2H, H_2_O molecule), ^13^C NMR (TMS, DMSO-d_6_ ) δ ppm: 169.42 (HC=N, azomethine), 150.94 (HC=N, pyridine ring), 141.98–140.52 (C=C pyridine ring C), 147.91 (C–O–C), 122.76–118.72 (Ar, C=C), 55.82 (OCH_3_).

Compound 11, yield: 58%, mp: 225°, IR (KBr) cm^-1^: 1615 (C=N, azomethine), 1279 (C–O, phenolic), 1006 (C=N, pyridine ring nitrogen), 589 (Mn–N), 518 (Mn–O), 3403, 987, 779 (–OH, H_2_O molecule). Compound 12, yield: 51%, mp: >300°, IR (KBr) cm^-1^: 1599 (C=N, azomethine), 1286 (C–O, phenolic), 1013 (C=N, pyridine ring nitrogen), 586 (Co–N), 516 (Co–O), 3394, 997, 765 (–OH, H_2_O molecule). Compound 13, yield: 68%, mp: 293°, IR (KBr) cm^-1^: 1605 (C=N, azomethine), 1298 (C–O, phenolic), 1350, 1009, 669 (C=N, pyridine ring nitrogen), 605 (Ni–N), 513 (Ni–O), 3379, 986, 776 (–OH, H_2_O molecule), 1631, 1405 (–COO, acetate group). Compound 14, yield: 83%, mp: 320°, IR (KBr) cm^-1^: 1620 (C=N, azomethine), 1287 (C-O, phenolic), 1012 (C=N, pyridine ring nitrogen), 605 (Cu–N), 517 (Cu–O), 3415, 980, 775 (–OH, H_2_O molecule), 1632, 1408 (–COO, acetate group). Compound 15, yield: 56%, mp: 254°, IR (KBr) cm^-1^: 1612 (C=N, azomethine), 1304 (C-O, phenolic), 1006 (C=N, pyridine ring nitrogen), 579 (Zn–N), 510 (Zn–O), 3410, 989, 787 (–OH, H_2_ O molecule), 1623, 1408 (–COO, acetate group). ^1^H NMR (TMS, DMSO-d_6_) δ ppm: 10.03 (s, 1H, HC=N, azomethine), 7.64–7.99 (d, 4H, Ar–H), 8.23–8.54 (s, pyridine, –HC=N), 3.40 (s, 3H, OCH_3_), 2.28 (s, 3H, CH_3_), 11.86 (s, 2H, SO_2_NH group), 4.67 (s, 2H, H_2_O molecule), ^13^C NMR (TMS, DMSO-d_6_) δ ppm: 172.26 (–HC=N, azomethine), 164.49 (HC=N, pyridine ring), 140.19 – 138.25 (C=C pyridine ring C), 148.17 (C–O–C), 124.27 – 119.58 (Ar, C= C), 56.15 (OCH_3_).

## RESULTS AND DISCUSSION

**Fig. 2:**
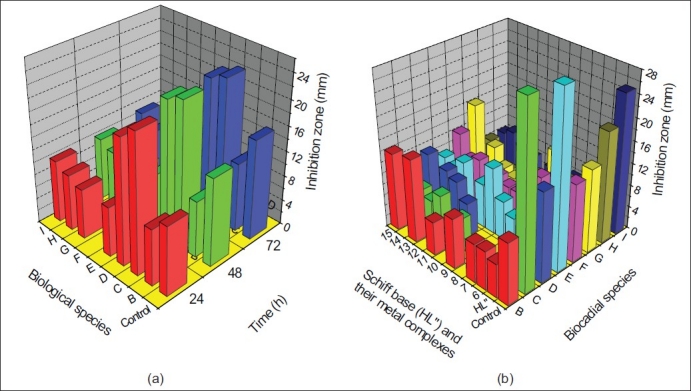
Biological activities of (a) mixed ligand complex (b) Schiff base complexes. (a) Mixed ligand complex (3) at 24, 48 and 72 h; (b) Schiff base complexes (HL", 6-10 and 11-15) at 24 h by well diffusion method (zone formation in mm); B- *Salmonella typhi*, C- *Pseudomonas aeruginosa*, D- *Escherichia coli*, E- *Vibrio parahaemolyticus*, F- *Aspergillus niger*, G- *Penicillium*, H- *Trichoderma virida and I- Saccrharomycies species*.

The zone of inhibition of the complexes against the growth of bacteria and fungi were given in Table 2 and Table 3, respectively. A representative graph is given in fig 2. From the table it is evident that the complexes (1-5) and (11-15) have higher antibacterial activity than the complexes (6-10). The complexes (1-5) and (6-10) shows higher antifungal activity than the complexes (11-15). In general, the synthesized metal complexes have higher biological activities compared to the free ligands. The increased inhibition activity of the metal complexes can be explained on the basis of Tweedy's chelation theory[14]. In metal complexes, on chelation the polarity of the metal ion will be reduced to a greater extent due to the overlap of the ligand orbital and partial sharing of the positive charge of the metal ion with donor groups. Further, it increases the delocalization of π- electrons over the whole chelate ring. The large ring size of 1,10-phenanthroline moiety makes the complexes more lipophillic[15]. This increased lipophillicity enhances the penetration of the metal complexes into lipid membranes and block the metal binding sites in the enzymes[16]. Metal complexes also disturb the respiration process of the cell and thus block the synthesis of proteins, which restricts further growth of the organisms. This enhancement in inhibiting the growth of bacteria and fungi can also be explained on the basis of their structure. The azomethine linkage and hetero aromatic moiety in the synthesized complexes exhibit extensive biological activities[17,18] due to increased liposolubility of the molecules in crossing cell membrane of the microorganism. The presence of electron donor group (-OCH_3_ ) in the complexes also plays a role in enhancing the inhibition activity. The antibacterial activity is found to be in the order; Control>(1-5)>(11-15)>(6-10)>HL'>HL". The antifungal activity is found to be in the order; Control>(6-10)>(1-5)>(11-15)>HL‘>HL".

**TABLE 2:** BACTERIAL ACTIVITIES OF THE SCHIFF BASE AND METAL COMPLEXES

Compound	Diameter of inhibition zone in mm for different microbial species											
	*Salmonella typhi*			*Pseudomonas aeruginosa*			*Escherichia coli*			*Vibrio parahaemolyticus*		
	24 h	48 h	72 h	24 h	48 h	72 h	24 h	48 h	72 h	24 h	48 h	72 h
HL'	7	8	8	8	9	9	10	11	12	8	9	9
HL"	6	7	7	7	8	8	-	-	-	7	8	8
1	9	10	10	9	11	12	9	11	11	9	10	13
2	-	-	-	14	15	16	-	-	-	8	8	11
3	9	9	11	22	23	23	20	22	22	8	9	9
4	-	9	10	8	10	10	-	-	-	10	10	10
5	9	10	10	-	-	-	12	12	14	10	11	12
6	7	7	8	-	-	-	-	-	-	6	7	9
7	7	7	8	-	-	-	-	-	-	6	7	9
8	-	-	-	-	-	-	-	-	-	5	10	10
9	9	8	7	-	-	-	-	-	-	7	8	9
10	-	-	-	6	7	7	-	-	-	12	13	14
11	6	8	8	-	-	-	6	7	8	8	9	9
12	-	-	-	8	9	9	9	9	10	-	-	-
13	15	15	16	6	7	7	10	12	13	10	10	11
14	-	-	-	7	8	9	-	-	-	8	8	9
15	14	15	17	11	12	12	11	14	14	9	11	12
Control	11	14	16	32	34	34	16	17	18	31	33	34

Each experiment was done in triplicate; [-, less active]; well diffusion method

**TABLE 3:** FUNGAL ACTIVITIES OF THE SCHIFF BASE AND METAL COMPLEXES

Compound	Diameter of inhibition zone in mm for different microbial species											
	*Aspergillus niger*			*Penicillium*			*Trichoderma virida*			*Saccharomyces cerevisiae*		
	24 h	48 h	72 h	24 h	48 h	72 h	24 h	48 h	72 h	24 h	48 h	72 h
HL'	5	6	6	5	6	6	8	8	9	8	9	9
HL"	6	6	6	-	6	8	6	7	7	4	5	6
1	-	-	-	9	9	10	13	14	14	12	12	13
2	9	9	10	9	10	11	-	-	-	8	8	9
3	-	9	9	8	8	9	9	9	9	10	10	11
4	10	11	11	9	10	10	12	14	14	9	10	10
5	-	10	10	-	9	10	12	12	13	10	11	12
6	10	12	13	10	11	12	8	9	9	8	11	12
7	12	14	16	15	16	16	8	8	8	6	7	8
8	10	12	12	-	-	-	4	4	8	6	8	10
9	7	9	13	-	-	-	4	6	6	-	-	-
10	7	8	16	13	15	16	6	10	10	6	8	8
11	8	8	10	6	8	8	4	4	4	-	-	-
12	-	-	-	7	8	8	-	-	-	6	6	6
13	9	12	12	10	10	11	4	4	4	-	-	-
14	8	8	9	-	-	-	-	-	-	9	9	10
15	12	13	14	16	18	19	8	12	12	8	8	8
Control	14	15	16	15	18	18	20	22	23	25	28	28

Each experiment was done in triplicate; [-, less active]; well diffusion method
